# Rethinking Crop Rotational Benefits Under Climate Change: Beyond the Growing Season

**DOI:** 10.1111/gcb.70012

**Published:** 2024-12-31

**Authors:** Monique E. Smith, Riccardo Bommarco, Giulia Vico

**Affiliations:** ^1^ Department of Ecology Swedish University of Agricultural Sciences Uppsala Sweden

## Abstract

Here, we provide a commentary on “Changes in the yield effect of the preceding crop in the US Corn Belt under a warming climate” recently published in Global Change Biology. Crop rotational diversity has recently been gaining interest for its role in climate change adaptation; however, the focus has been on climatic conditions in the growing season . This study uses data from over a wide area and an important cropping system to demonstrate that the benefits of a different preceding crop for both corn and soybean depended on both growing season and non‐growing conditions.

In large regions, agriculture has become industrialized and highly specialized. This has resulted in much higher yields compared to pre‐industrialization, but the contribution of industrialized agriculture to environmental degradation, climate change, and biodiversity loss has often been overlooked. Crop rotational diversity is gaining renewed momentum in both research and policy as a way to reverse some of this damage. Indeed, rotating diverse crops in the field is not a new practice. It dates back to the Roman empire and is still common in organic farms, to break pest cycles and improve soil fertility and thus reduce the need for environmentally damaging agrochemicals. This practice has also been shown to improve other ecosystem services, such as carbon sequestration, nutrient cycling, water regulation, and biodiversity, without compromising yields (Smith et al. 2023; Tamburini et al. 2020). More recently, crop rotations are also gaining interest for their role in climate change adaptation.

Analyses of long‐term field experiments have demonstrated that diverse crop rotations can mitigate yield loss under adverse climatic conditions (Bowles et al. 2020). Adding a single non‐cereal crop to a rotation could counterbalance the negative effects of detrimental climatic conditions, such as anomalous warm, wet or dry conditions (Costa et al. 2024). To make generalizations over time and space, following the approach previously assessed by Kluger, Owen, and Lobell (2022), Zhou et al. (2024) gathered yield maps generated using satellite imagery across the US Corn Belt, covering a timespan of 19 years. They then contrasted the yields from monocultures of corn and soybean with yields from rotated corn and soybean. This approach has the advantage of allowing a wide coverage—impossible to achieve via field experiments—but is limited to regions, crops, and cropping systems for which data are available. For example, Zhou et al. (2024) were limited to the dominant rotation in the region, 2‐year corn‐soybean rotations. Based on this, the rotation diversity effect cannot be distinguished from the preceding crop effect. However, the advantages of shifting from a monoculture to a simple rotation can be quantified under a wide range of climates and climatic conditions.

Benefits of diverse rotations have been attributed to improved soil structure and increased soil organic matter content that enhances water and nutrient retention (Bennett et al. 2012; Renwick et al. 2021). Similarly, the preceding crop can affect root architecture and thus ability to withstand water stress (Cope et al. 2024). Additionally, incorporating diverse crops in rotations disrupts cycles of pests, pathogens, and weeds. This was demonstrated in corn‐soybean rotations whereby corn rhizospheres were enriched with beneficial bacteria, fungi, and mycoviruses, and suppressed in fungal pathogens compared with continuous corn (Liu et al. 2024). What is most novel about Zhou et al. (2024) was linking the preceding crop effects to the climatic conditions, including those outside the growing season (GS).

Climatic conditions greatly influence crop yields and, in general, ecosystem processes. Thus far, the focus on determining the effects of climatic conditions, possibly in interaction with practices like rotational diversity, has been on the GS. However, at least some of the mechanisms involved in rotational benefits can also be impacted by non‐growing season (NGS) climatic conditions. For example, warmer NGS temperatures can increase nutrient cycling and reduce the amount of nutrient available for the subsequent crop. Breaking pest cycles may also be more important when NGS temperatures increase and improve pest overwintering conditions. Indeed, Zhou et al. (2024) show that benefits of a different preceding crop for both corn and soybean depended on both GS and NGS conditions. The effects were not trivial. The corn rotational benefit decreased with increasing NGS temperature and precipitation as well as GS temperature but increased with GS precipitation when GS temperature was high. Conversely, for soybean, the benefits of corn as a preceding crop decreased with increasing GS and NGS temperature. When combined with climate projections, these results offer the opportunity to estimate changes in rotational benefits under future climates. By 2051–2070, benefits of soybean as a preceding crop for corn yields were predicted to be reduced, mostly due to increasing NGS temperature and rainfall, although with some regional variations due to current climatic conditions. Mechanistically, the reduced benefit could be related to either higher NGS temperatures increasing nutrient cycling and increased NGS precipitation reducing the nutrient benefits of the soybean in rotation due to leaching. Alternatively, climate warming in the GS could improve soil nitrogen availability for continuous corn, particularly in the northern states which would also reduce the rotational benefit. Differently from corn, the benefits to soybean of corn as a preceding crop depended primarily on GS temperature and were projected to increase in the northern US Corn Belt and slightly decline in the south. This geographical pattern might be due to warming enhancing pest pressure in current cooler regions, calling for diverse rotations to break pest cycles, but exceeding the thermal suitability of more southern locations. Despite the decline in rotational benefit in corn, future county‐level yields of rotated corn and soybean were concluded to remain higher compared with the current ones, when considering the projected future rotational benefit in combination with the 2‐year rotation being fully implemented.

Based on the likely mechanisms involved in crop rotational diversity effects, it is expected that management practices beyond rotation diversification interact with climatic conditions in defining rotational benefits. Zhou et al. (2024) attempted to quantify these effects over the US Corn Belt by relating preceding crop benefits to state‐level data on pesticide and fertilizer use and data on the crops lost to damage. For corn, preceding crop benefits decreased with fertilizer use, suggesting that at least some of the rotational benefits are to be ascribed to enhanced nitrogen availability (Kluger, Owen, and Lobell 2022). Preceding crop benefits decreased also with pesticide use, supporting the role of rotational diversity for breaking pest cycles. However, the relationship of preceding crop effects with pesticide use was on average weak in soybean and location specific, with higher benefits in the southern states which also had the highest pesticide use.

Overall, Zhou et al. (2024) points to some very important conclusions about rotational benefits under climate change in soybean‐corn rotation, including the role of NGS climatic conditions. At the same time, it reminds us of important remaining knowledge gaps. Mechanisms behind the crop rotational benefits are multiple and still only partially understood. The relative role of the mechanisms at play remains difficult to quantify, in part due to this requiring multiple detailed, long‐term experiments, which are clearly logistically difficult. Importantly, the rotations used in Zhou et al. (2024) have the lowest diversity possible to qualify as a rotation, alternating only two crops. It has been shown in long‐term trials that the largest rotation benefits happen with between three and four crops in the rotation and can be even higher in organic farms (Smith et al. 2023). The benefits of rotational diversity for climate change adaptation are thus likely grossly underestimated. Indeed, further diversifying rotations, including additional functional types beyond cereal and legume, such as ley or broadleaves, could lead to cereal yields higher under detrimental conditions than those under beneficial conditions (Costa et al. 2024). The relative importance and interactions between the preceding crop effect and the whole rotational diversity remains to be assessed. Finally, the uncertainties in estimating the rotational benefits and how they are affected by climatic conditions compound with uncertainties in climate projections when aiming at quantifying the climate change adaptation. In summary, Zhou et al. (2024) also shows the importance of the often‐overlooked NGS over a wide area and an important cropping system. At the same time, it shows how limited our understanding still is. This is a serious drawback when aiming at mainstreaming crop rotational diversity as climate change adaptation.

## Author Contributions

**Monique E. Smith:** conceptualization, writing – original draft, writing – review and editing. **Riccardo Bommarco:** writing – review and editing. **Giulia Vico:** writing – review and editing.

## Conflicts of Interest

The authors declare no conflicts of interest.

## Data Availability

The authors have nothing to report.
